# Ações contra a Covid-19 na População com Síndrome de Down

**DOI:** 10.36660/abc.20200685

**Published:** 2020-11-01

**Authors:** Giorgia Castilho Russo, Nathalia Bernardes, Natália Rezende Baraldi, Denise Jeanine Berlinger Saraiva, Kátia De Angelis, Carla Janice Baister Lantieri, José Francisco Saraiva

**Affiliations:** 1 Serviço Nacional de Aprendizagem Comercial São Paulo São PauloSP Brasil Serviço Nacional de Aprendizagem Comercial São Paulo, São Paulo, SP -Brasil; 2 Universidade Nove de Julho Laboratório de Fisiologia Translacional São PauloSP Brasil Universidade Nove de Julho - Laboratório de Fisiologia Translacional, São Paulo, SP - Brasil; 3 Universidade São Judas Tadeu Laboratório do Movimento Humano São PauloSP Brasil Universidade São Judas Tadeu - Laboratório do Movimento Humano, São Paulo, SP - Brasil; 4 Pontifícia Universidade Católica de Campinas CampinasSP Brasil Pontifícia Universidade Católica de Campinas, Campinas, SP - Brasil; 5 Prefeitura de São Paulo Prefeitura Municipal de Campinas CampinasSP Brasil Prefeitura de São Paulo - Prefeitura Municipal de Campinas, Campinas, SP - Brasil; 6 Universidade Federal de São Paulo São PauloSP Brasil Universidade Federal de São Paulo, São Paulo, SP - Brasil; 7 Universidade Municipal de São Caetano do Sul São Caetano do SulSP Brasil Universidade Municipal de São Caetano do Sul, São Caetano do Sul, SP - Brasil; 8 Sociedade Brasileira de Cardiologia Rio de JaneiroRJ Brasil Sociedade Brasileira de Cardiologia, Rio de Janeiro, RJ – Brasil

**Keywords:** Coronavírus, Pandemia, COVID-19, Saúde Pública/organização e administração, Síndrome de Down, Infecções, Doenças Cardiovasculares, Autonomia Pessoal, Qualidade de Vida, Alimentação Saudável, Citocinas

A Organização Mundial de Saúde (OMS) declarou, em 30 de janeiro de 2020, que o surto da doença do novo coronavírus (COVID-19) constitui uma Emergência de Saúde Pública de Importância Internacional — o nível mais alto de alerta da Organização, de acordo com o Regulamento Sanitário Internacional. Em 11 de março de 2020, a COVID-19 foi declarada pandemia.^1^ O fato de a letalidade da COVID-19 estar associada a comorbidades comuns gerou grande preocupação entre profissionais que trabalham com pessoas com Síndrome de Down (SD). A prevalência de doenças cardiovasculares em pessoas com SD é de 40-50%,^2^^,^^3^ e eles também têm maior propensão ao sobrepeso e à obesidade; além disso, pacientes com SD apresentam alterações nas vias aéreas que facilitam a infecção pelo vírus,^4^ o que pode piorar os efeitos da COVID-19. Além disso, crianças com SD são mais suscetíveis a infecções, devido a alterações na regulação de citocinas, enquanto os adultos frequentemente apresentam aumento dos biomarcadores pró-inflamatórios. Essas alterações podem ter impacto nas doenças anatômicas dos pacientes e aumentar a prevalência de condições inflamatórias crônicas e a mortalidade por sepse**.**^5^

Atualmente, a única estratégia reconhecida para prevenir a infecção pelo coronavírus da síndrome respiratória aguda grave 2 (*severe acute respiratory syndrome coronavirus* 2, SARS-CoV-2) é evitar a exposição ao vírus.^4^ O isolamento social foi recomendado por vários estados brasileiros com a finalidade de separar as pessoas saudáveis daquelas com suspeita de COVID-19 ou que tiveram contato com casos suspeitos ou confirmados de COVID-19. Vale destacar que a socialização é um aspecto muito importante do tratamento de pessoas com SD e que contribui para uma melhor qualidade de vida e autonomia;^3^ portanto, o isolamento social representa um grande desafio para essa população.

Considerando esses aspectos dos pacientes com SD, considerou-se crucial uma resposta rápida contra a COVID-19 relativa a essa população. Três associações brasileiras [Fundação Síndrome de Down (FSD), Pontifícia Universidade Católica de Campinas (PUC-Campinas) e Sociedade Brasileira de Cardiologia (SBC)], se reuniram para produzir materiais de apoio para jovens com SD e suas famílias. O atendimento às pessoas com SD deve ser interdisciplinar e necessita de diferentes especialidades;^3^ as instituições reuniram profissionais e estudantes das áreas de medicina, jornalismo, educação básica, nutrição, psicologia, terapia ocupacional e farmácia. Ademais, a iniciativa incluiu o apoio voluntário de editores, produtores, desenvolvedores e animadores para elaborar materiais práticos, rápidos e comunicativos sobre os principais pontos de atenção para prevenir a COVID-19 com uma linguagem apropriada a essa população. Vale mencionar o importante apoio recebido pela PUC-Campinas; a universidade implementou o núcleo de extensão da sua missão e gerou conhecimento para ajudar a comunidade a desenvolver soluções para os seus problemas.

O material foi produzido em conformidade com a campanha “We decide” (Nós decidimos), proposta pela organização de suporte global Down Syndrome International no Dia Internacional da Síndrome de Down. As estratégias de comunicação empregadas nesse projeto envolvem diálogo, acolhimento e a participação do paciente no processo terapêutico, os quais são considerados estratégias eficientes para pessoas com SD. Primeiramente, criou-se um vídeo animado para explorar a percepção visual e as habilidades visuais de aprendizagem. A mensagem foi reiterada por escrito, explorando a habilidade de leitura. Um discurso claro e direto considerou o uso de aparelhos auditivos, pois essa população pode apresentar perda de acuidade auditiva.^6^ Essa estratégia priorizou o fornecimento de informações necessárias para capacitar a população com SD na realização da prevenção primária de saúde e no reconhecimento das ações necessárias para promover sua segurança.

Como estratégia secundária, os jovens com SD foram orientados a produzir vídeos que fornecessem informações sobre práticas de prevenção da COVID-19, explorando o desejo dos jovens de se comunicar e socializar, assim como sua disposição em modelar o comportamento do seu ambiente social.^7^ Essa etapa resultou em vários vídeos curtos, compartilhados com a mídia, nos quais a população com SD ilustrou práticas de lavagem de mãos, distanciamento social, alimentação saudável e atividades físicas, além de descrever quais eram as atividades favoritas dos jovens em casa durante a pandemia; essas medidas contribuíram para promover a saúde mental e física durante esse período. Também exibimos um vídeo preparado pelos tutores, a fim de garantir o fornecimento de informações completas e concisas para auxiliar aqueles com menor escolaridade. Todos os vídeos foram também adaptados para os usuários da Língua Brasileira de Sinais (Libras), o que expandiu o seu público, e podem ser acessados no link: https://www.facebook.com/watch/Previna-Covid-19-101822971493321/

É consenso entre as equipes responsáveis pelo cuidado de pessoas com SD que investimentos em saúde, educação e inclusão social resultam em uma maior qualidade de vida e níveis mais elevados de autonomia. Portanto, estratégias de apoio devem estar centradas no fornecimento de informações de forma tanto direta para pessoas com SD como indireta para seus familiares e cuidadores; ao enfocar o papel e a autonomia dessas pessoas para compartilhar o cuidado, também estamos promovendo a saúde da família.^3^ Assim, foi elaborada uma carta de apoio aos pais e aos cuidadores para complementar os materiais de apoio e foi disponibilizada nas redes sociais, podendo ser acessada no link: https://drive.google.com/drive/u/0/folders/1meN5KdHjeYzMkbsGRDM61ufJMjEg09Ml

É Importante enfatizar que todos os materiais preparados pela equipe se basearam em medidas de atenção à saúde e hábitos saudáveis. Considerando que a SD é um fator de risco para complicações da COVID-19,^4^ o objetivo foi oferecer informações concisas e, especialmente, simplificadas que possam ser rapidamente implementadas, destacando a importância do distanciamento social e da higiene pessoal. Focamos em explicar a importância da prevenção primária: ilustrar a maneira correta de desinfetar as mãos, a importância de não compartilhar itens pessoais, do distanciamento social e das condutas adequadas caso seja necessário sair de casa.^7^ Além disso, os principais sintomas de COVID-19^4^^–^^7^ foram explicados, e os pacientes foram aconselhados a pedir a ajuda de familiares ou cuidadores caso reconheçam alguns desses sintomas. O guia também orientou aqueles que têm contato direto com pessoas com SD sobre como distinguir a gravidade dos casos suspeitos de COVID-19, orientado e permitindo que eles solicitem ajuda especializada ao mesmo tempo em que evitam exposição desnecessária. Além disso, foram reforçadas boas práticas de saúde, tais como conformidade com o calendário de vacinação para pessoas com SD, a importância da saúde mental e a adoção de medidas de saúde preventiva em períodos de pandemia, assim como a orientação para usar máscara.

Com relação aos hábitos de saúde, bons hábitos de alimentação são um aspecto importante do estilo de vida saudável das pessoas com SD. Algumas pessoas com SD podem ter predisposição à obesidade, e uma dieta inapropriada podem comprometer seu estilo de vida e induzir alterações em seus sistemas imunes. Considerando esses aspectos, nosso material se baseou no Guia Alimentar para a População Brasileira, o qual incentiva o consumo de alimentos frescos e desaconselha o consumo de alimentos ultraprocessados. A presença da família no domicílio também reforça outra importante orientação do guia, que é a comensalidade. As pessoas são incentivadas a comer e cozinhar junto com seus familiares, promovendo o consumo de alimentos frescos. Ademais, há evidências de que o SARS-CoV-2 pode permanecer ativo em alguns tipos de superfícies por longos períodos de tempo: um estudo relatou que vírus como SARS-CoV-2, SARS-CoV e MERS-CoV poderiam permanecer em superfícies por até nove dias. Assim, a desinfecção de superfícies (tais como alimentos e embalagens ao chegar do supermercado e durante a preparação de refeições) deve ser realizada frequentemente**.**^2^^,^^7^

Outro aspecto importante a ser considerado durante o isolamento social é a ocorrência de comportamento sedentário e de baixos níveis de atividade física, os quais são fatores de risco para pessoas com SD. De fato, um estilo de vida sedentário aumenta a suscetibilidade a infecções virais e o desenvolvimento de fatores de risco para doenças cardiovasculares e metabólicas^8^ que são mais frequentes em pessoas com SD, tais como obesidade, hipertensão e diabetes.^9^ Portanto, é extremamente importante que as pessoas com SD evitem o comportamento sedentário (sentar no sofá ou ficar deitado na cama por longos períodos, ou até mesmo perder tempo com aparelhos eletrônicos) e pratiquem atividades físicas^8^ durante o isolamento social. Recomenda-se atividade física aeróbica de intensidade moderada (20 a 60 minutos) de 3 a 7 vezes por semana; essas atividades podem incluir caminhada ou corrida, saltos, futebol, ou dança. Também devem ser incluídos nessa rotina exercícios de força envolvendo diferentes grupos musculares (utilizando acessórios alternativos como faixas elásticas, garrafas pet como halteres, e até mesmo o peso do próprio corpo), com pelo menos 8-12 repetições (intensidade moderada), 2 vezes por semana.^10^**,**^11^

As atividades da vida diária, tais como limpar a casa ou o quintal, subir escadas ou brincar com um animal de estimação também são importantes para manter as pessoas com SD fisicamente ativas e integradas. As atividades físicas devem ser realizadas preferencialmente em um espaço seguro e ventilado. É essencial buscar atividades físicas agradáveis, tais como brincar com uma boa ou dançar, aumentando assim a adesão e os benefícios fisiológicos e psicossociais dessa prática.

A sociedade atual está cada vez mais consciente da importância de valorizar a diversidade humana e oferecer oportunidades iguais para que pessoas com deficiências exerçam seu direito de viver na comunidade. Portanto, a forma de comunicação proposta nesta ação é direcionada exclusivamente às necessidades das pessoas com SD com enfoque em uma demanda social por entender a COVID-19. Em meio à pandemia, diante da necessidade de fornecer informações precisas e seguras para esse grupo de risco, uma equipe multidisciplinar com ações transdisciplinares desenvolveu estratégias para fortalecer o cuidado de adolescentes e adultos com SD. O objetivo principal foi manter aspectos da atenção à saúde (incluindo hábitos alimentares e de sono, imunização e atividade física), assim como a autonomia para as atividades instrumentais e básicas da vida diária, tais como autocuidado, socialização, escolaridade e orientação vocacional.

O protagonismo das pessoas com SD na promoção da prevenção da COVID-19 contribuiu para a sua inclusão social e para a quebra do estigma de limitações carregado pela população com SD. Por fim, a educação e o apoio da família são aspectos importantes no ensino das práticas de atenção à saúde, identificando os hábitos e o estilo de vida recomendados como abordagens fundamentais para a prevenção da COVID-19 na população com SD.
