# Supplementary material for: Ações contra a Covid-19 na População com Síndrome de Down
Source: Arq Bras Cardiol. 2020 Nov 1;115(5):939–41. [Article in Portuguese] doi: 10.36660/abc.20200685 (PMC8452218; doi:10.36660/abc.20200685)
Supplement: Translation

# Translation: Actions Against Covid-19 in the Down Syndrome Population

**Authors:** Giorgia Castilho Russo, Nathalia Bernardes, Natália Rezende Baraldi, Denise Jeanine Berlinger Saraiva, Kátia De Angelis, Carla Janice Baister Lantieri, José Francisco Saraiva

**Affiliations:** 1 São PauloSP Brazil Serviço Nacional de Aprendizagem Comercial São Paulo, São Paulo, SP -Brazil; 2 São PauloSP Brazil Universidade Nove de Julho - Laboratório de Fisiologia Translacional, São Paulo, SP - Brazil; 3 São PauloSP Brazil Universidade São Judas Tadeu - Laboratório do Movimento Humano, São Paulo, SP - Brazil; 4 CampinasSP Brazil Pontifícia Universidade Católica de Campinas, Campinas, SP - Brazil; 5 CampinasSP Brazil Prefeitura de São Paulo - Prefeitura Municipal de Campinas, Campinas, SP - Brazil; 6 São PauloSP Brazil Universidade Federal de São Paulo, São Paulo, SP - Brazil; 7 São Caetano do SulSP Brazil Universidade Municipal de São Caetano do Sul, São Caetano do Sul, SP - Brazil; 8 Rio de JaneiroRJ Brazil Sociedade Brasileira de Cardiologia, Rio de Janeiro, RJ – Brazil

**Keywords:** Coronavirus, Pandemics, COVID-19, Public Health/organization and administration, Down Syndrome, Infections, Cardiovascular Diseases, Personal Autonomy, Quality of Life, Diet, Healthy, Cytokines

The World Health Organization (WHO) declared, on January 30, 2020, that the outbreak of the new coronavirus disease (COVID-19) constituted a Public Health Emergency of International Importance — the organization's highest alert level as per its International Health Regulations. On March 11, 2020, COVID-19 was declared a pandemic.^1^ The fact that the lethality of COVID-19 is associated with common comorbidities has caused great concern among professionals who work with people with Down Syndrome (DS). The prevalence of cardiovascular diseases in people with DS is 40-50%,^2^^,^^3^ and they are also more prone to overweight and obesity; moreover, patients with DS present changes in airways that facilitate infection by the virus,^4^ which can worsen the effects of COVID-19. In addition, children with DS are more susceptible to infections due to changes in cytokines regulation, while adults frequently display increased proinflammatory biomarkers. These changes can impact the patients' anatomical disorders and increase the incidence of chronic inflammatory conditions and mortality by sepsis.^5^

Currently, the only recognized strategy to prevent infection by the severe acute respiratory syndrome coronavirus 2 (SARS-CoV-2) is avoiding exposure to the virus.^4^ Social isolation was advised by several Brazilian states with the aim of separating healthy people from those with suspected COVID-19, or who had contact with suspected or confirmed COVID-19 cases. It is worth noting that socialization is a very important aspect of the treatment of people with DS that contributes to a better quality of life and autonomy;^3^ therefore, social isolation represents a major challenge for this population.

Considering these aspects of patients with DS, a quick response against COVID-19 regarding this population was considered crucial. Three Brazilian associations (Fundação Síndrome de Down [FSD]; Pontifícia Universidade Católica de Campinas [PUC-Campinas]; and the Brazilian Society of Cardiology [SBC]) came together to produce support material for young people with DS and their families. The care of people with DS must be interprofessional and requires different specialties;^3^ the institutions thus brought together professionals and students of the areas of medicine, journalism, basic education, nutrition, psychology, occupational therapy, and pharmacy. Moreover, the initiative included the voluntary support of editors, producers, designers, and animators to build practical, fast, and communicative material addressing the main points of attention for preventing COVID-19 with an appropriate language for this population. The important support received by PUC-Campinas is worth mentioning; the university implemented the extension branch of its mission and generated knowledge to help the community develop solutions for its problems.

The material was produced in line with the “We decide” campaign, proposed by the Down Syndrome International global support on World Down Syndrome Day. Communication strategies employed in this project involved dialogue, as well as the reception and participation of the patient in the therapeutic process, which are considered efficient approaches for people with DS. First, an animated video was developed to explore visual awareness and visual learning skills. The message was reaffirmed in writing, exploring the ability to read. A clear and direct discourse considered the use of hearing aids, since this population may present some loss of auditory acuity.^6^ This strategy prioritized providing the necessary information to empower the population with DS to perform primary health prevention and recognize the necessary actions to promote their safety.

As a second strategy, young people with DS were instructed to produce videos that provided information regarding COVID-19 prevention practices, exploring the desire to communicate and socialize as well as their willingness to model the behavior of their social environment.^7^ This stage resulted in several short videos, shared with the media, in which the population with DS illustrated practices of hand washing, social distancing, healthy eating, physical activities, and described what were their favorite activities at home during the pandemic; these measures contributed to promote mental and physical health during this period. We also broadcasted a video prepared by tutors, aiming to guarantee complete and concise information to assist those with less education. All videos were further adapted for Brazilian sign language (Libras) users, which expanded their public, and can be accessed at the link: https://www.facebook.com/watch/Previna-Covid-19-101822971493321/

It is a consensus among teams working in the care of people with DS that investments in health, education, and social inclusion result in a higher quality of life and higher levels of autonomy. Therefore, support strategies must be focused on providing information both directly to people with DS and indirectly to their family and caregivers; by focusing on their role and autonomy to share care, we are also promoting family health.3 A support letter for parents and caregivers was thus designed to complete the support materials and was made available on social networks; it can be accessed at the link: https://drive.google.com/drive/u/0/folders/1meN5KdHjeYzMkbsGRDM61ufJMjEg09Ml

It is important to emphasize that all materials prepared by the team were based on health care measures and healthy habits. Considering that DS is a risk factor for complications of COVID-19,^4^ the aim was to offer concise and, especially, easy information that could be quickly adopted, highlighting the importance of social distancing and personal hygiene. We focused on explaining the importance of primary prevention: illustrating the correct way to disinfect hands, the importance of not sharing personal items, social distancing, and adequate attitudes in case they needed to leave their residence.^7^ In addition, the main symptoms of COVID-19^4^^–^^7^ were explained and patients were advised to ask for help from family members or caregivers in case they recognized any of them. The guidebook also instructed those in direct contact with people with DS to distinguish the severity of suspected COVID-19 cases, guiding and enabling them to request specialized help while avoiding unnecessary exposure. In addition, good health practices were reinforced, such as compliance to the vaccination schedule for people with DS, the importance of mental health, and the adoption of preventive health measures in pandemic periods, as well as the instruction to wear a face mask.

Regarding health habits, good eating habits are an important aspect of the healthy lifestyle of people with DS. Some people with DS may have a predisposition to obesity and an inappropriate diet could compromise their lifestyle and induce changes to their immune systems. Considering these aspects, our material was based on the Food Guide for the Brazilian Population, which encourages the consumption of fresh foods and warns against the consumption of ultra-processed foods. The presence of the family in the household also reinforces another important orientation of the guide, which is commensality. People are encouraged to eat and cook along with their families, promoting the consumption of fresh foods. In addition, there is evidence that SARS-CoV-2 may remain active on some types of surfaces for long periods of time: one study reported that viruses like SARS-CoV-2, SARS-CoV, and MERS-CoV could remain on surfaces for up to nine days. Thus, the disinfection of surfaces (such as food and packages when arriving from the supermarket and during meal preparation) must be performed frequently.^2^^,^^7^

Another important aspect to be considered during social isolation is the occurrence of sedentary behavior and low levels of physical activity, which are risk factors for people with DS. In fact, a sedentary lifestyle increases susceptibility to viral infections and the development of risk factors for cardiovascular and metabolic diseases^8^ that are more frequent in people with DS, such as obesity, hypertension, and diabetes.^9^ Thus, it is extremely important for people with DS to avoid sedentary behavior (sitting on the couch or lying in bed for long periods, or even fiddling with electronic devices) and to practice physical activities^8^ during social isolation. Moderate-intensity aerobic physical activity (20 to 60 minutes) is recommended 3 to 7 times a week; this may include walking or running, jumping, playing soccer, or dancing. Strength exercises involving different muscle groups (making use of alternative accessories, such as elastic bands, pet bottles as dumbbells, and even the weight of the body itself), with at least 8-12 repetitions (moderate intensity), 2 times a week should also be included in this routine.^10^**,**^11^

The activities of daily living, such as cleaning the house or yard, climbing stairs, or playing with a pet are also important to keep people with DS physically active and integrated. Physical activities should preferably be performed in a safe and ventilated space. It is essential to look for pleasurable physical activities, such as playing with a ball or dancing, thus increasing the adherence and physiological and psychosocial benefits of this practice.

The current society is increasingly aware of the importance of valuing human diversity and offering equal opportunities for people with disabilities to exercise their right to live in the community. Therefore, the form of communication proposed in this action was exclusively aimed at the needs of people with DS, focusing on a social demand for understanding COVID-19. In the midst of the pandemic, faced with the need to bring accurate and safe information to this risk group, a multidisciplinary team with transdisciplinary actions developed strategies to reinforce the health care of adolescents and adults with DS. The ultimate aim was to maintain aspects of health care (including eating and sleeping habits, immunization, and physical activity), as well as the autonomy for basic and instrumental activities of daily living such as self-care, socialization, schooling, and vocational guidance.

The protagonism of people with DS in promoting COVID-19 prevention has contributed to their social inclusion and to breaking the stigma of limitations carried by the population with DS. Finally, education and family support are important aspects when teaching health care practices, relating the recommended habits and lifestyle as fundamental approaches for the prevention of COVID-19 in the population with DS.
